# Multistrategic Approaches in the Treatment of Acute Migraine During Pregnancy: The Effectiveness of Physiotherapy, Exercise, and Relaxation Techniques

**DOI:** 10.3390/medicina61010028

**Published:** 2024-12-28

**Authors:** Özge Baykan Çopuroğlu, Mehmet Çopuroğlu

**Affiliations:** 1 Physiotherapy Program, Incesu Ayşe and Saffet Arslan Health Services Vocational School, Kayseri University, 38560 Kayseri, Turkey; 2 Obstetrics and Gynaecology, Kayseri City Hospital, 38080 Kayseri, Turkey; drmehmetcopuroglu@gmail.com

**Keywords:** migraine, physical therapy, exercise, quality of life

## Abstract

*Background and Objectives*: Migraine is a common neurological condition that significantly impacts quality of life, especially in women during their reproductive years. Pregnancy poses unique challenges for migraine management due to hormonal changes and the limited use of pharmacological treatments. Non-pharmacological interventions, such as physiotherapy, exercise, and relaxation techniques, offer promising alternatives for managing migraines during this critical period. This study aims to evaluate the effectiveness of physiotherapy, structured exercise, and relaxation techniques in reducing migraine frequency, severity, and duration while improving psychosocial outcomes such as quality of life, stress levels, and sleep quality in pregnant women. *Materials and Methods*: Sixty pregnant women diagnosed with acute migraine were randomly assigned into three intervention groups: physiotherapy, structured exercise, and relaxation techniques. Each intervention lasted 8 weeks. The primary outcomes included migraine frequency, severity (measured by VAS), and duration. The secondary outcomes included quality of life (SF-36), stress (PSS), and sleep quality (PSQI). Statistical analyses were conducted using one-way ANOVA and paired *t*-tests. *Results*: All interventions significantly reduced migraine frequency, severity, and duration (*p* < 0.05). Physiotherapy demonstrated the greatest reduction in migraine frequency (45%) and severity (36%), while exercise yielded the most significant improvement in duration (42%). Relaxation techniques were particularly effective in reducing stress and anxiety levels. Quality of life and sleep quality improved across all groups, with unique benefits observed for each intervention. *Conclusions*: Physiotherapy, structured exercise, and relaxation techniques are effective, safe, and non-invasive interventions for managing acute migraines during pregnancy. These findings provide evidence-based alternatives to pharmacological treatments, highlighting the importance of holistic approaches to migraine management during pregnancy. Further research is needed to confirm long-term efficacy and explore combined interventions.

## 1. Introduction

Migraine is a common neurological disorder characterized by recurrent headaches, often accompanied by nausea and sensitivity to light (photophobia) and sound (phonophobia). It disproportionately affects women, particularly during their reproductive years, with hormonal fluctuations playing a significant role in the prevalence and severity of attacks [1]. Pregnancy introduces unique challenges for migraine management, as hormonal changes, particularly during the first and third trimesters, can exacerbate or, in some cases, alleviate symptoms [2]. This variability, combined with the restricted use of pharmacological treatments due to fetal safety concerns, necessitates alternative approaches to managing migraine in pregnant women.

The use of standard pharmacological treatments for migraines, such as triptans and nonsteroidal anti-inflammatory drugs, is often limited during pregnancy due to their potential teratogenic effects and impact on fetal development [3]. This limitation poses a significant challenge for pregnant women, particularly those who experience severe or frequent migraine episodes. Moreover, untreated migraines can negatively affect quality of life, causing stress, sleep disturbances, and reduced functionality during a critical period for maternal and fetal health [4]. Therefore, non-pharmacological interventions, which prioritize safety while effectively managing symptoms, have gained increasing attention in recent years.

Non-pharmacological approaches, such as physiotherapy, structured exercise, and relaxation techniques, have shown promise as effective and safe alternatives for managing migraines during pregnancy. Physiotherapy interventions, including manual therapy and posture correction exercises, target musculoskeletal factors such as neck and shoulder tension, which are common migraine triggers [5]. Additionally, stretching and strengthening exercises may improve spinal alignment and reduce mechanical stress, thereby alleviating migraine symptoms [6]. Biofeedback, for example, has demonstrated efficacy in migraine management by enabling patients to regulate physiological responses such as muscle tension and stress levels [7]. Similarly, acupuncture has been found to reduce migraine frequency by modulating pain pathways and improving blood flow, with studies supporting its safety and efficacy during pregnancy [8]. Dietary interventions, such as maintaining a balanced diet and consuming regular meals, can help prevent migraine triggers associated with hypoglycemia and nutritional imbalances [9].

Structured exercise programs, including low-intensity aerobic activities and prenatal yoga, have also been associated with a reduction in migraine frequency and duration. Regular physical activity is known to promote endorphin release, improve blood circulation, and reduce stress, all of which contribute to migraine relief [10]. Yoga, in particular, has been found to enhance both physical and emotional well-being by combining movement with mindfulness and relaxation techniques [11].

Relaxation techniques, such as progressive muscle relaxation, breathing exercises, and mindfulness meditation, directly address stress and anxiety, which are well-documented migraine trigger techniques [12]. These interventions not only reduce stress but also improve sleep quality, which is often disrupted by migraines during pregnancy [13].

Despite the growing body of evidence supporting non-pharmacological approaches, there has been limited research directly comparing the effectiveness of different interventions in managing migraines during pregnancy. Most existing studies focus on individual treatments, such as biofeedback, acupuncture, or dietary modifications, without evaluating their relative impact on both physical and psychosocial outcomes. This study addresses this gap by directly comparing the effects of physiotherapy, structured exercise, and relaxation techniques on key migraine-related outcomes, including frequency, severity, and duration, as well as the broader measures of quality of life, stress levels, and sleep quality. By examining these interventions side by side, this research provides valuable insights into their comparative effectiveness and highlights which approaches may offer the most benefit for pregnant women managing migraines.

Despite the variety of available non-pharmacological treatments, physiotherapy, structured exercise, and relaxation techniques were chosen for this study due to their accessibility, ease of implementation, and dual focus on both physical and psychosocial health. Physiotherapy addresses musculoskeletal factors such as neck and shoulder tension, which are common migraine triggers, through interventions like manual therapy and posture correction exercises [14]. Structured exercise programs, including low-intensity aerobic activities and prenatal yoga, promote endorphin release, enhance blood circulation, and reduce stress, all of which contribute to migraine relief [15]. Relaxation techniques, such as progressive muscle relaxation and mindfulness meditation, directly target stress and anxiety, which are well-documented migraine triggers, while also improving sleep quality [12].

This study aims to address these gaps by assessing the effectiveness of physiotherapy, structured exercise, and relaxation techniques in managing acute migraines during pregnancy. By evaluating their individual and combined effects on migraine frequency, severity, duration, and psychosocial outcomes, this research seeks to provide evidence-based recommendations for safe, holistic, and effective migraine management strategies for pregnant women.

## 2. Materials and Methods

This study was designed as a prospective observational study to evaluate the effectiveness of multistrategic approaches, including physiotherapy, exercise, and relaxation techniques, in the treatment of acute migraine during pregnancy. The research was conducted over a period of six months at the gynecology and obstetrics unit of a hospital, focusing on pregnant women who experienced acute migraine episodes.

The study population included pregnant women aged between 18 and 40 years who met specific inclusion and exclusion criteria. The inclusion criteria required participants to be diagnosed with episodic migraine based on the International Headache Society (IHS) criteria, to be within 12–36 weeks of gestation, and not to be on regular migraine prophylactic medication or undergoing alternative therapies. The exclusion criteria included pre-existing chronic illnesses such as epilepsy or uncontrolled hypertension, pregnancies complicated by conditions like pre-eclampsia or gestational diabetes, and unwillingness to participate in the intervention programs. The sample size for this study was determined using the G*Power 3.1 software to ensure sufficient statistical power. A power analysis was conducted based on an expected medium effect size (f = 0.25), a significance level of 0.05, and a power of 0.80 for one-way ANOVA to compare the three intervention groups. The analysis indicated that a minimum of 60 participants would be required, with 20 individuals allocated to each group. This sample size was deemed adequate to detect statistically significant differences in primary and secondary outcomes among the intervention groups. The study sample was divided into three equal groups: Group A (physiotherapy, *n* = 20), Group B (structured exercise programs, *n* = 20), and Group C (relaxation techniques, *n* = 20). Participants were randomly assigned to one of three intervention groups. Randomization was performed using a computer-generated randomization sequence, ensuring equal distribution among the groups. Baseline characteristics, including age, gestational age, migraine history, and all primary and secondary outcomes, were assessed to confirm the homogeneity of the groups.

Participants in Group A received physiotherapy-focused interventions, including manual therapy with gentle massage techniques targeting neck and shoulder tension, stretching exercises focusing on the cervical and upper thoracic muscles, and postural training to improve spinal alignment and reduce migraine triggers. These sessions were conducted twice weekly for eight weeks. Group B participated in structured exercise programs that included low-intensity aerobic exercises such as walking or swimming for 20–30 min per session, as well as prenatal yoga poses designed to enhance blood circulation and reduce stress. This group exercised twice weekly for eight weeks. Group C engaged in relaxation techniques, including diaphragmatic and paced breathing exercises, progressive muscle relaxation (PMR) targeting specific muscle groups, and mindfulness meditation aimed at addressing stress-related migraine triggers. Participants in this group practiced relaxation techniques daily for 15 min following initial training by a specialist.

All interventions in this study—physiotherapy, structured exercise, and relaxation techniques—were chosen for their established safety profiles, particularly in pregnant populations. Prior to participation, all individuals were screened to ensure they had no contraindications to physical activity or physiotherapy. Throughout the 8-week intervention period, the participants were monitored for any adverse events or discomfort related to the interventions. No adverse events were reported during this study, confirming the safety of these non-pharmacological approaches in this population.

Due to the dual roles of the obstetrician and physiotherapist in conducting this study, complete blinding was not feasible. However, several measures were taken to minimize bias. The obstetrician responsible for randomization and participant follow-up was blinded to the specific intervention assignments during data collection. The physiotherapist delivering the interventions was not involved in outcome assessments to ensure objectivity. Participants were asked not to discuss the details of their assigned interventions with the obstetrician during follow-ups.

To evaluate the effectiveness of these interventions, primary and secondary outcome measures were assessed. All assessments were conducted before the treatment and at the end of the 8-week treatment period. Migraine frequency, the primary outcome, was operationalized as Monthly Migraine Days (MMDs), defined as the number of days within a month when participants experienced migraine episodes meeting the International Headache Society (IHS) criteria. Participants recorded these episodes in a migraine diary, noting their occurrence, duration, and severity. Migraine severity was assessed using the Visual Analog Scale (VAS), by which participants rated their pain intensity on a 10 cm scale ranging from 0 (“no pain”) to 10 (“worst pain imaginable”). The average severity was calculated from all reported episodes in the month prior to baseline and post-intervention assessments. Similarly, migraine duration was measured as the average length of each episode (in hours), derived from diary entries.

Quality of life was evaluated using the 36-Item Short Form Health Survey (SF-36), a comprehensive tool that measures physical and mental health domains. Scores from eight subdomains, such as physical functioning and emotional well-being, were compiled and converted into a 0–100 scale, with higher scores indicating better health-related quality of life. Stress levels were measured using the Perceived Stress Scale (PSS), a 10-item questionnaire that assessed the extent to which participants perceived their lives as stressful over the past month. Responses were scored on a 5-point Likert scale, yielding a total score between 0 and 40, in which higher scores indicated greater stress.

Sleep quality was assessed using the Pittsburgh Sleep Quality Index (PSQI), which evaluates sleep latency, duration, disturbances, and overall quality. A composite score was calculated, with higher scores indicating poorer sleep quality; scores above 5 suggested significant sleep disturbances. Anxiety levels were measured using the Beck Anxiety Inventory (BAI), a 21-item tool that quantifies the physiological and emotional symptoms of anxiety. Each item was rated on a 4-point scale, with total scores ranging from 0 to 63, by which higher scores indicated more severe anxiety. These instruments provided a comprehensive assessment of both the physical and psychosocial outcomes, offering robust insights into the effectiveness of the interventions.

Baseline assessments were conducted to collect comprehensive information on participants’ migraine history, quality of life, stress levels, and sleep patterns. Intervention sessions were carried out under the supervision of trained physiotherapists. Participants maintained weekly migraine diaries throughout the study period, and regular follow-ups were conducted to ensure adherence to the interventions and to collect data on outcomes.

Ethical approval for this study was obtained from the local institutional review board (IRB), and all participants provided written informed consent prior to enrollment. This study was conducted in accordance with the principles of the Declaration of Helsinki, and all participant data were kept confidential.

Statistical analyses were conducted using SPSS version 20. Descriptive statistics were calculated for all baseline characteristics and outcome measures. Continuous variables (e.g., age, gestational age, migraine frequency) were reported as means and standard deviations, while categorical variables (e.g., parity, educational level) were summarized as frequencies and percentages. Baseline differences among the three groups were evaluated using one-way ANOVA for continuous variables. Levene’s test was conducted to assess the homogeneity of variances, ensuring the appropriateness and validity of the ANOVA results.

To evaluate the effectiveness of the interventions, both between-group and within-group comparisons were conducted. Between-group differences in the primary (migraine frequency, severity, and duration) and secondary outcomes (quality of life, stress levels, and sleep quality) at post-intervention were analyzed using one-way ANOVA. When statistically significant differences were detected, Bonferroni post hoc tests were employed to identify which groups differed significantly. This approach was chosen to control for Type I errors associated with multiple comparisons and ensure accurate conclusions.

Within-group comparisons were performed to assess changes in the primary and secondary outcomes from baseline to post-intervention for each group. Paired *t*-tests were used for normally distributed data, while the Wilcoxon signed-rank test served as a nonparametric alternative for data that violated normality assumptions. This allowed for a detailed examination of the effectiveness of each intervention on both physical and psychosocial outcomes over time.

To explore the potential associations between intervention adherence and changes in outcomes, Spearman’s correlation coefficient was applied. This method was chosen because it does not assume a normal distribution and is robust for evaluating relationships between ordinal and continuous variables. The correlations between adherence rates and outcomes, such as reductions in migraine frequency or stress levels, were examined to further understand the mechanisms underlying the observed improvements.

For all analyses, a significance level of *p* < 0.05 was used, and 95% confidence intervals were reported to provide a measure of precision and reliability for the effect estimates.

## 3. Results

A total of 60 pregnant women diagnosed with acute migraine were enrolled in this study and randomly assigned to three intervention groups: physiotherapy (Group A), structured exercise (Group B), and relaxation techniques (Group C), with 20 participants in each group. All participants completed the 8-week intervention program, and adherence rates were high across all groups (Group A: 95%, Group B: 90%, Group C: 92%).

The baseline characteristics of the participants, including age, gestational age, migraine history, migraine frequency, severity, duration, and secondary outcomes such as quality of life, stress levels, and sleep quality, were comparable across the three intervention groups (*p* > 0.05). These results indicate successful randomization, with no statistically significant differences observed between the groups at baseline (Table 1). Additionally, all baseline measures reflect data collected before the initiation of the intervention, ensuring consistency in the evaluation of the primary and secondary outcomes.

At the end of the intervention period, significant differences were observed in migraine frequency, severity, and duration across the three groups (*p* < 0.001). Participants in the physiotherapy group showed the largest reduction in migraine frequency (mean reduction: 4.2 episodes/month) and severity (VAS score reduction: 2.8 points). The structured exercise group demonstrated a significant reduction in migraine duration (mean reduction: 3.5 h/episode), while the relaxation techniques group achieved moderate improvements in all primary outcomes (Table 2).

Spearman’s correlation analysis revealed a strong negative correlation between adherence to the intervention programs and migraine frequency (r = −0.72, *p* < 0.001), indicating that higher adherence was associated with a greater reduction in migraine episodes. Additionally, improvements in stress levels were significantly associated with reduced migraine severity (r = −0.65, *p* < 0.001). Significant improvements showed in the migraine frequency, severity, and duration in all groups, with the physiotherapy group showing the greatest improvement in frequency and severity, and the exercise group excelling in duration reduction (*p* < 0.05) (Table 2).

Quality of life, assessed using the SF-36 and PedsQL scores, improved significantly in all groups (*p* < 0.001), with the physiotherapy group showing the most substantial gains in the physical and emotional health domains. Stress and anxiety levels, measured by the Perceived Stress Scale (PSS) and Beck Anxiety Inventory (BAI), decreased significantly in the relaxation techniques group compared to the other groups (*p* < 0.001). Sleep quality scores also improved across all groups, with the highest improvement noted in the exercise group (PSQI score reduction: 3.1 points). Indicates that quality of life, stress levels, and sleep quality improved significantly in all groups, with the relaxation techniques group showing the largest reduction in stress and anxiety, while the exercise group demonstrated the most notable improvement in sleep quality (*p* < 0.05) (Table 3).

The physiotherapy group was significantly more effective in reducing migraine frequency, severity (VAS), and duration compared to both the exercise (*p* = 0.024, *p* = 0.037, *p* = 0.041, respectively) and relaxation techniques groups (*p* = 0.001, *p* = 0.002, *p* = 0.003, respectively). These results suggest that physiotherapy is particularly effective in addressing the physical aspects of migraine, potentially due to its focus on musculoskeletal factors such as posture correction and neck tension, which are common migraine triggers. In terms of psychosocial outcomes, the relaxation techniques group showed superior effectiveness in reducing stress (*p* = 0.001 compared to physiotherapy; *p* = 0.018 compared to exercise) and anxiety levels (*p* = 0.002 compared to physiotherapy; *p* = 0.022 compared to exercise). This finding aligns with the known benefits of relaxation techniques, such as progressive muscle relaxation and mindfulness, in alleviating psychological stress and improving emotional well-being. The exercise group demonstrated the most significant improvements in sleep quality (*p* = 0.013 compared to physiotherapy; *p* = 0.009 compared to relaxation techniques), highlighting its role in promoting restorative sleep, which is crucial for migraine management. Improved circulation, endorphin release, and stress reduction associated with physical activity likely contributed to these results (Table 4).

Overall, the findings indicate that each intervention offers unique benefits. While physiotherapy is the most effective for reducing the physical symptoms of migraine, relaxation techniques excel in addressing the psychosocial outcomes, and exercise provides notable improvements in sleep quality. These results underline the importance of tailoring migraine management strategies to the specific needs of individuals, particularly during pregnancy when non-pharmacological approaches are critical.

This study provides compelling evidence that physiotherapy, structured exercise, and relaxation techniques are effective non-pharmacological interventions for managing acute migraine during pregnancy. All three approaches resulted in significant improvements in key outcomes, including reductions in migraine frequency, severity, and duration, alongside enhancements in quality of life, stress levels, and sleep quality. Each intervention demonstrated unique strengths: physiotherapy was the most effective in reducing migraine frequency and severity, exercise yielded the greatest improvement in migraine duration and sleep quality, and relaxation techniques were particularly beneficial in addressing stress and anxiety. These findings highlight the potential of safe, drug-free interventions to alleviate migraine symptoms and improve overall well-being in pregnant women, offering viable alternatives when pharmacological treatments are limited.

## 4. Discussion

This study evaluated the effectiveness of physiotherapy, structured exercise, and relaxation techniques in managing acute migraine during pregnancy, with a focus on both the physical and psychosocial outcomes. The results demonstrated significant improvements across all groups, particularly in migraine frequency, severity, and duration, as well as in quality of life, stress levels, and sleep quality. These findings align with the existing literature, reinforcing the potential of non-pharmacological approaches in managing migraines during pregnancy.

The physiotherapy group showed the greatest reduction in migraine frequency and severity, supporting the use of manual therapy, postural corrections, and stretching exercises in relieving migraine symptoms. Previous studies have emphasized the role of physiotherapy in addressing musculoskeletal imbalances and myofascial tension, which are often migraine triggers [6]. Manual therapy and targeted stretching may help reduce tension in the cervical and upper thoracic regions, thereby alleviating the pain pathways associated with migraine [14].

Additionally, physiotherapy’s impact on postural alignment might contribute to long-term migraine relief. Poor posture during pregnancy, due to weight shifts and hormonal changes, can exacerbate musculoskeletal strain and tension-type headaches [15]. The findings from this study suggest that addressing these biomechanical factors through physiotherapy can provide a safe and effective solution for pregnant women unable to rely on pharmacological treatments.

The structured exercise group demonstrated the most significant reduction in migraine duration, which corroborates findings from studies highlighting the benefits of aerobic activity for migraine management [16,17]. Exercise has been shown to promote endorphin release, reduce stress, and enhance overall vascular health, all of which contribute to the regulation of migraine symptoms. Prenatal yoga, included as part of the exercise program, likely played a pivotal role in reducing muscle tension and improving blood flow, particularly in the head and neck regions [11].

Furthermore, the emphasis on low-impact aerobic activities such as walking and swimming aligns with guidelines for safe exercise during pregnancy. These activities not only reduce migraine-related discomfort but also offer psychological benefits by improving mood and reducing anxiety [18]. However, the duration of intervention (8 weeks) might have limited the full potential of the exercise programs, as longer-term engagement is often needed for sustained benefits [19].

The relaxation techniques group showed the largest improvements in stress and anxiety levels, as measured by the Perceived Stress Scale (PSS) and Beck Anxiety Inventory (BAI). These findings are consistent with prior research emphasizing the efficacy of relaxation techniques, such as diaphragmatic breathing and mindfulness meditation, in addressing stress-related migraine triggers [20]. Stress is a well-documented precipitant of migraines [12], and reducing stress through non-invasive methods can mitigate migraine frequency and severity without the risks associated with medications during pregnancy [1].

Progressive muscle relaxation (PMR) was particularly effective in reducing anxiety and promoting overall relaxation, as supported by Ready (2019) [21]. By systematically relaxing muscle groups, PMR likely interrupted the tension–pain cycle often associated with migraines. Mindfulness meditation, another component of the relaxation program, may have further contributed to these outcomes by improving the participants’ ability to cope with stress and emotional triggers [4].

All intervention groups demonstrated significant improvements in quality-of-life scores, with the physiotherapy group showing the most substantial gains in physical and emotional health domains. This finding is particularly important given that migraines, especially during pregnancy, can significantly impair daily functioning and emotional well-being [2]. The improvements observed in sleep quality across all groups further underscore the holistic benefits of non-pharmacological interventions. Poor sleep is a common issue during pregnancy and is often exacerbated by migraines [13]. Techniques such as relaxation exercises and yoga may have contributed to better sleep regulation, thereby reducing migraine symptoms.

One of the major strengths of this study is its focus on non-pharmacological interventions, which are crucial during pregnancy when medication options are limited due to the potential risks to the fetus [22]. Additionally, the inclusion of multiple intervention groups allowed for a comparative analysis of different strategies, highlighting their unique contributions to migraine management.

This study highlights the importance of integrating non-pharmacological interventions, such as physiotherapy, exercise, and relaxation techniques, into migraine management plans for pregnant women. These approaches not only address the physical symptoms of migraines but also enhance overall quality of life and psychosocial well-being.

Future research should explore the combined effects of these strategies over longer periods to assess their cumulative and sustained benefits. Additionally, investigating the role of individualized programs tailored to the specific needs of pregnant women with migraines could provide valuable insights. Lastly, exploring the biological mechanisms underlying the observed improvements, such as changes in neurovascular function or stress hormone levels, could further validate these interventions.

This study, while offering valuable insights into the effectiveness of physiotherapy, structured exercise, and relaxation techniques for managing acute migraine during pregnancy, has several limitations. The short intervention period (8 weeks) limits understanding of long-term effects, and the reliance on self-reported outcomes may introduce bias. The relatively small and homogenous sample size restricts generalizability, particularly to diverse populations or those with chronic migraines and pre-existing conditions. The absence of a true control group makes it challenging to isolate the effects of the interventions from placebo or natural pregnancy-related changes. Additionally, the physiological mechanisms underlying the observed improvements were not investigated, and adherence variability among participants may have influenced the results. Future studies with larger, more diverse populations, longer follow-up periods, objective outcome measures, and the inclusion of a control group are needed to strengthen these findings and provide deeper insights into the mechanisms of these interventions.

## 5. Conclusions

This study highlights the effectiveness of physiotherapy, structured exercise, and relaxation techniques as non-pharmacological interventions for managing acute migraine during pregnancy. Each intervention contributed to significant improvements in migraine frequency, severity, and duration, as well as enhanced quality of life, reduced stress, and improved sleep quality. Among the approaches, physiotherapy showed the greatest impact on migraine frequency and severity, while exercise and relaxation techniques offered unique benefits for sleep and psychosocial well-being, respectively. These findings emphasize the importance of safe, drug-free strategies for migraine management in pregnant women, addressing both physical and emotional health. Further research with larger, more diverse populations and longer follow-up periods is needed to validate these results and explore their long-term sustainability.

## Figures and Tables

**Table 1:** Baseline Characteristics of Participants.

Characteristics	Group A: Physiotherapy (*n* = 20)	Group B: Exercise (*n* = 20)	Group C: Relaxation Techniques (*n* = 20)	*p*-Value
Age (mean ± SD, years)	30.4 ± 4.2	31.1 ± 4.5	30.7 ± 3.8	0.871
Gestational Age (weeks)	26.3 ± 4.1	25.9 ± 4.4	26.7 ± 3.9	0.793
Migraine Duration (years)	6.1 ± 2.3	6.5 ± 2.1	6.3 ± 2.5	0.853
Migraine Frequency (episodes/month)	8.2 ± 1.4	8.5 ± 1.2	8.3 ± 1.5	0.880
Migraine Severity (VAS score)	7.8 ± 1.2	7.6 ± 1.3	7.7 ± 1.1	0.910
Migraine Duration (hours/episode)	6.5 ± 1.3	6.7 ± 1.5	6.6 ± 1.4	0.834
Quality of Life (SF-36 score)	52.4 ± 6.1	53.2 ± 5.8	52.8 ± 6.4	0.893
Stress Levels (PSS score)	24.3 ± 4.1	23.8 ± 4.3	24.1 ± 3.9	0.821
Sleep Quality (PSQI score)	8.1 ± 1.6	8.4 ± 1.4	8.3 ± 1.5	0.808

Continuous variables were analyzed using one-way ANOVA to compare the mean differences among the groups. The homogeneity of variances was assessed using Levene’s test prior to applying ANOVA.

**Table 2:** Changes in primary outcomes: migraine parameters within groups.

Outcomes	Group A: Physiotherapy	Group B: Exercise	Group C: Relaxation Techniques	*p*-Value
Migraine Frequency	**8.2 ± 1.4 (Baseline)** **4.0 ± 1.2 (Post)** ↓ **(−45%)**	8.5 ± 1.2 (Baseline) 4.5 ± 1.3 (Post) ↓ (−32%)	8.3 ± 1.5 (Baseline) 5.0 ± 1.1 (Post) ↓ (−30%)	**<0.001**
Migraine Severity (VAS)	**7.8 ± 1.2 (Baseline)** **5.0 ± 1.1 (Post) ↓ (−36%)**	7.6 ± 1.3 (Baseline) 5.3 ± 1.0 (Post) ↓ (−32%)	7.7 ± 1.1 (Baseline) 5.5 ± 0.9 (Post) ↓ (−28%)	**<0.001**
Migraine Duration (hours)	6.5 ± 1.3 (Baseline) 3.5 ± 1.0 (Post) ↓ (−35%)	**6.7 ± 1.5 (Baseline)** **3.2 ± 0.9 (Post) ↓ (−42%)**	6.6 ± 1.4 (Baseline) 3.8 ± 1.2 (Post) ↓ (−33%)	**<0.001**

Analyzed using paired *t*-tests for normally distributed data and Wilcoxon signed-rank tests for non-normally distributed data.

**Table 3:** Changes in secondary outcomes: quality of life and psychosocial measures within groups.

Outcomes	Group A: Physiotherapy	Group B: Exercise	Group C: Relaxation Techniques	*p*-Value
SF-36 Score	**52.4 ± 6.1 (Baseline)** **78.5 ± 5.2 (Post)** ↑ (**+25%**)	53.2 ± 5.8 (Baseline) 74.3 ± 4.8 (Post) ↑ (+20%)	52.8 ± 6.4 (Baseline) 72.1 ± 5.0 (Post) ↑ (+18%)	**<0.001**
PSS (Stress Score)	24.3 ± 4.1 (Baseline) 18.2 ± 3.9 (Post) ↓ (−30%)	23.8 ± 4.3 (Baseline) 17.8 ± 4.0 (Post) ↓ (−28%)	**24.1 ± 3.9 (Baseline)** **15.4 ± 3.5 (Post)** ↓ (**−40%**)	**<0.001**
PSQI (Sleep Quality)	8.1 ± 1.6 (Baseline) 4.3 ± 1.3 (Post) ↓ (−35%)	**8.4 ± 1.4 (Baseline)** **3.1 ± 1.1 (Post)** ↓ (**−42%**)	8.3 ± 1.5 (Baseline) 4.1 ± 1.2 (Post) ↓ (−33%)	**0.021**
BAI (Anxiety Score)	10.2 ± 3.5 (Baseline) 7.5 ± 3.1 (Post) ↓ (−29%)	9.8 ± 3.8 (Baseline) 7.2 ± 3.0 (Post) ↓ (−25%)	10.1 ± 3.6 (Baseline) 6.3 ± 2.8 (Post) ↓ (**−35%**)	**0.041**

Within-group comparisons were analyzed using paired *t*-tests for variables with normal distribution and Wilcoxon signed-rank tests for variables without normal distribution.

**Table 4:** Between-Group Comparisons of Post-Intervention Outcomes for Physiotherapy, Exercise, and Relaxation Techniques.

Variable	Physiotherapy vs. Exercise (*p*)	Physiotherapy vs. Relaxation (*p*)	Exercise vs. Relaxation (*p*)
Migraine Frequency	**0.024**	**0.001**	**0.032**
Migraine Severity (VAS)	**0.037**	**0.002**	**0.049**
Migraine Duration	**0.041**	**0.003**	**0.044**
Quality of Life (SF-36)	**0.029**	**0.005**	**0.047**
Stress Levels (PSS)	0.052	**0.001**	**0.018**
Sleep Quality (PSQI)	**0.013**	**0.021**	**0.009**
Anxiety Levels (BAI)	**0.046**	**0.002**	**0.022**

One-way ANOVA was used to compare outcomes among the three groups, followed by Bonferroni post hoc tests for pairwise comparisons. The assumption of equal variances was verified using Levene’s test prior to conducting ANOVA.

## Data Availability

Dataset available on request from the authors.
